# Progression of *ampC* amplification during *de novo* amoxicillin resistance development in *E. coli*

**DOI:** 10.1128/mbio.02982-24

**Published:** 2024-12-20

**Authors:** Luyuan Nong, Martijs Jonker, Wim de Leeuw, Meike T. Wortel, Benno ter Kuile

**Affiliations:** 1 Biology and Microbial Food Safety, Swammerdam Institute for Life Sciences, University of Amsterdam, Amsterdam, the Netherlands; 2 RNA Biology & Applied Bioinformatics, Swammerdam Institute for Life Sciences, University of Amsterdam, Amsterdam, the Netherlands; University of Texas Health Science Center, School of Public Health, Houston, Texas, USA

**Keywords:** AmpC, beta-lactamase, gene amplification, resistance evolution, antimicrobial, IS1 element, DNA mutations

## Abstract

Beta-lactam antibiotics are the most applied antimicrobials in human and veterinarian health care. Hence, beta-lactam resistance is a major health problem. Gene amplification of AmpC beta-lactamase is a main contributor to *de novo* β-lactam resistance in *Escherichia coli*. However, the time course of amplification and the accompanying DNA mutations are unclear. Here, we study the progression of *ampC* amplification and *ampC* promoter mutations during the evolution of resistance induced by stepwise increasing amoxicillin concentrations. *AmpC* promoter mutations occurred by day 2, while the approximately eight-fold amplification occurred after more than 6 days of amoxicillin exposure. The combination of the amplification and the promoter mutations increased the *ampC* mRNA level by an average factor of 200 after 22 days. An IS*1* insertion is identified in the amplification junction after resistance induction in the wild type (WT) and the *ampC* gene complementation strain (CompA), but not in ∆*ampC*, suggesting that the amplification depends on mobile genetic element transposition. In order to elucidate the correlation between gene mutations and *ampC* amplification, the DNA mutations acquired during resistance evolution by the WT, ∆*ampC*, and CompA were analyzed. Compared to evolved ∆*ampC*, several resistance-causing mutations are absent in evolved WT, while more mutations accumulated in stress response. The amoxicillin-resistant ∆*ampC* did not show amplification of the fragment around the original *ampC* position but exhibited a large duplication or triplication at another position, suggesting the essential role of the duplicated genes in resistance development.

**IMPORTANCE:**

Amoxicillin is the most used antimicrobial against bacterial infections. DNA fragments containing *ampC* are amplified upon prolonged and stepwise increasing exposure to amoxicillin, causing resistance. These *ampC*-containing fragments have been identified in extended-spectrum beta-lactamase plasmids, which are considered the main cause of beta-lactam resistance. In this study, we document the time course of two important factors for *ampC* transcription enhancement, *ampC* amplification and *ampC* promoter mutations, during *de novo* amoxicillin resistance evolution. We propose that the transposon IS*1* contributes to the amplification *ampC* region, that the sigma factor 70 regulates *ampC* overexpression, and that these combined form the backbone of a putative mechanism for *ampC* amplification.

## INTRODUCTION

Although alternative antibiotics are available (1, 2), beta-lactam antimicrobials remain the most commonly used antibiotics for human infection treatment (3). Exposure to sublethal levels of antibiotics is the main reason for resistance emergence (4). When the effectiveness of antibiotics is reduced, exposure to sublethal levels during antibiotic treatment is unavoidable (5). The risk of non-lethal concentrations occurs, especially in the veterinary sector, when livestock is treated with antibiotics added to water or feed. Under long-term exposure, antimicrobial resistance may accumulate in the microbiome, and this resistance can be transmitted to human pathogens through horizontal gene transfer.

AmpC beta-lactamases (AmpC) are widely distributed cephalosporinases causing beta-lactam degradation (6). The *ampC* gene can be encoded by both chromosomal and plasmid DNAs. Unlike extended-spectrum beta-lactamases (ESBLs), which are generally considered the main reason of the beta-lactam resistance (7), the overexpression of *ampC* is underestimated as cause of resistance. In *Escherichia coli*, the chromosomal *ampC* gene is poorly expressed in the wild type, as it lacks the transcription activator, AmpR (8). However, after laboratory evolution of amoxicillin resistance, the transcription of *ampC* is enhanced more than 100-fold compared to the naive strain mainly because of two reasons: *ampC* promoter mutations and gene multiplication (9).

The *AmpC* gene is controlled by a weak promoter (P*_ampC_*), which has three main elements, a −10 box, a −35 box, and an attenuator (10). The mutations C > T in the −10 box at position −11 and T > A in the −35 box at −32 are conservative, increasing AmpC production by 21- and 7-fold, respectively (10). In addition, *ampC* gene amplification plays a considerable role in its transcriptional enhancement (11). Chromosomal *ampC* amplification increases resistance (12). This amplification is a RecA-independent event occurring in tandem (13, 14). Duplicated antibiotic resistance genes can transfer horizontally in microbial communities, with mobile genetic elements serving as a vehicle (15). A chromosomal DNA fragment containing the *ampC* gene was amplified from strains made resistant to amoxicillin by exposure to stepwise increasing concentrations and isolated with plasmid isolation techniques (16). This fragment can be exchanged between *E. coli* strains by horizontal gene transfer. A similar *E. coli* chromosomal fragment harboring *ampC* and two nearby genes can be identified in several ESBL plasmids isolated in broiler production (17). This suggests that *ampC* amplification may occur in *E. coli* developing beta-lactam resistance, and that, subsequently, the fragment is incorporated in ESBL plasmids.

In addition to inactivation of the antibiotic through AmpC, various other molecular mechanisms can contribute to beta-lactam resistance, including active efflux pumps, decreased influx, and target site modification (18). Multiple point mutations were demonstrated to directly contribute to resistance, such as mutations in the efflux pump AcrAB-TolC (19) and the outer membrane porin OmpC/OmpF (20). Besides, some mutations that alter metabolism also confer to resistance (21, 22). For example, a mutation in the 2-oxoglutarate dehydrogenase (*sucA*) gene raised carbenicillin resistance through lower basal respiration, thereby avoiding metabolic toxicity and reducing lethality.

To understand the competitive, synergistic, and epistatic effects of DNA mutations associated with *ampC* amplification, this study investigates the time course of mutations in the chromosome and *ampC* amplification. Using the *de novo* development of amoxicillin resistance in *E. coli* as a model, this study addresses five questions: (i) What is the time course and pattern of *ampC* amplification? (ii) Is the amplified *ampC* fragment always the same, or can the length vary? (iii) Is the *ampC* copy number the primary factor determining the AmpC activity? (iv) If the *ampC* gene is removed, does the amplification of the fragment around it still occur? (v) Is the pattern of mutations accompanying the development of resistance different in the presence and absence of *ampC*? Answering these questions provides insights into the complex dynamics of beta-lactam resistance development and the role of *ampC* amplification in this process.

## RESULTS

### Relationship between AmpC and amoxicillin resistance

**Fig 1:**
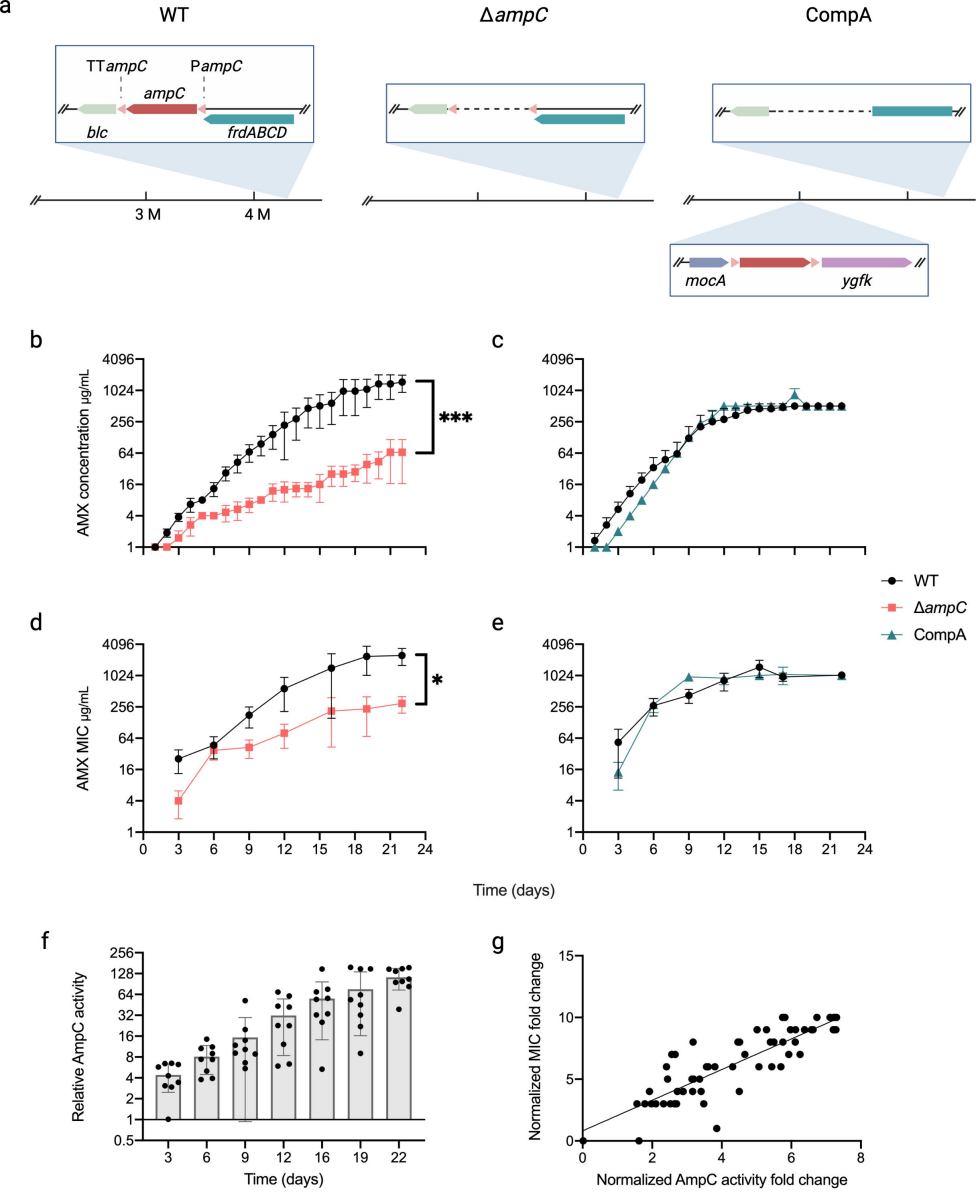
Role of AmpC in the evolution of amoxicillin resistance. (**a**) Genetic differences between the wild type and the ∆*ampC* and CompA mutants. Gene direction is indicated with arrows and genes by different colors. Promoter (P) and terminator (TT) are shown as triangles. (**b and c**) Amoxicillin concentration in the cultures. (**d and e**) MIC for amoxicillin of evolved strains. Statistical significance was determined with Wilcoxon signed-rank test, ***P < 0.001, *P < 0.05. (**f**) Measurement of AmpC activity in the wild type. (**g**) Relationship between MIC and AmpC activity (*R*^2^ = 0.730).

To investigate the role of AmpC in amoxicillin resistance, wild-type *E. coli* (WT), an *ampC* knockout mutant derived from it (∆*ampC*), and an *ampC* complementation strain (CompA) created by moving *ampC* to a different location in the chromosome were made resistant by growing them at stepwise increasing sublethal amoxicillin concentrations (Fig. 1). The CompA strain was produced by moving the DNA sequence of *E. coli* between bp 4,367,403 and 4,368,895, which contains the promoter, the reading frame, and the terminator of the *ampC* gene, to the 3,009,241 position, which is between the *mocA* and *ygfK* genes (Fig. 1a). The *ampC* promoter sequence contains part of the *frdD* gene. The deletion of the *ampC* expression cassette inevitably renders the *frdABCD* operon inoperable. The minimal inhibitory concentration (MIC) of the naïve WT for amoxicillin is 4 mg/L in minimal medium and 8 mg/L in rich medium (LB) as compared to 2 mg/L for ∆*ampC* in both mediums. The growth of CompA is hampered in the minimal medium. The MIC of CompA is the same as that of WT in the LB medium, 8 mg/L.

The acquisition of resistance by ∆*ampC* occurs at a significantly slower rate compared to WT. Correspondingly, the MIC reached at least 1024 mg/L in the WT and only between 16 and 64 mg/L in ∆*ampC* (Fig. 1d). There was no difference in the adaptive speed between WT and CompA (Fig. 1c and e). The AmpC activity was determined using the chromogenic substrate nitrocefin in the presence of intact live cells, as the enzyme functions as an ectoenzyme (9). Compared to the activity of the naive wild type, the activities encountered in the evolved WT increased by a factor of 39–156 in the final incubations (Fig. 1f). The log_2_-transformed fold change of MIC and AmpC activity exhibited a linear relationship with *R*^2^ = 0.730 (Fig. 1g). This indicates that *de novo* amoxicillin resistance in *E. coli* can be largely attributed to the increase in AmpC activity.

### *ampC* gene amplification during the evolution of resistance

**Fig 2:**
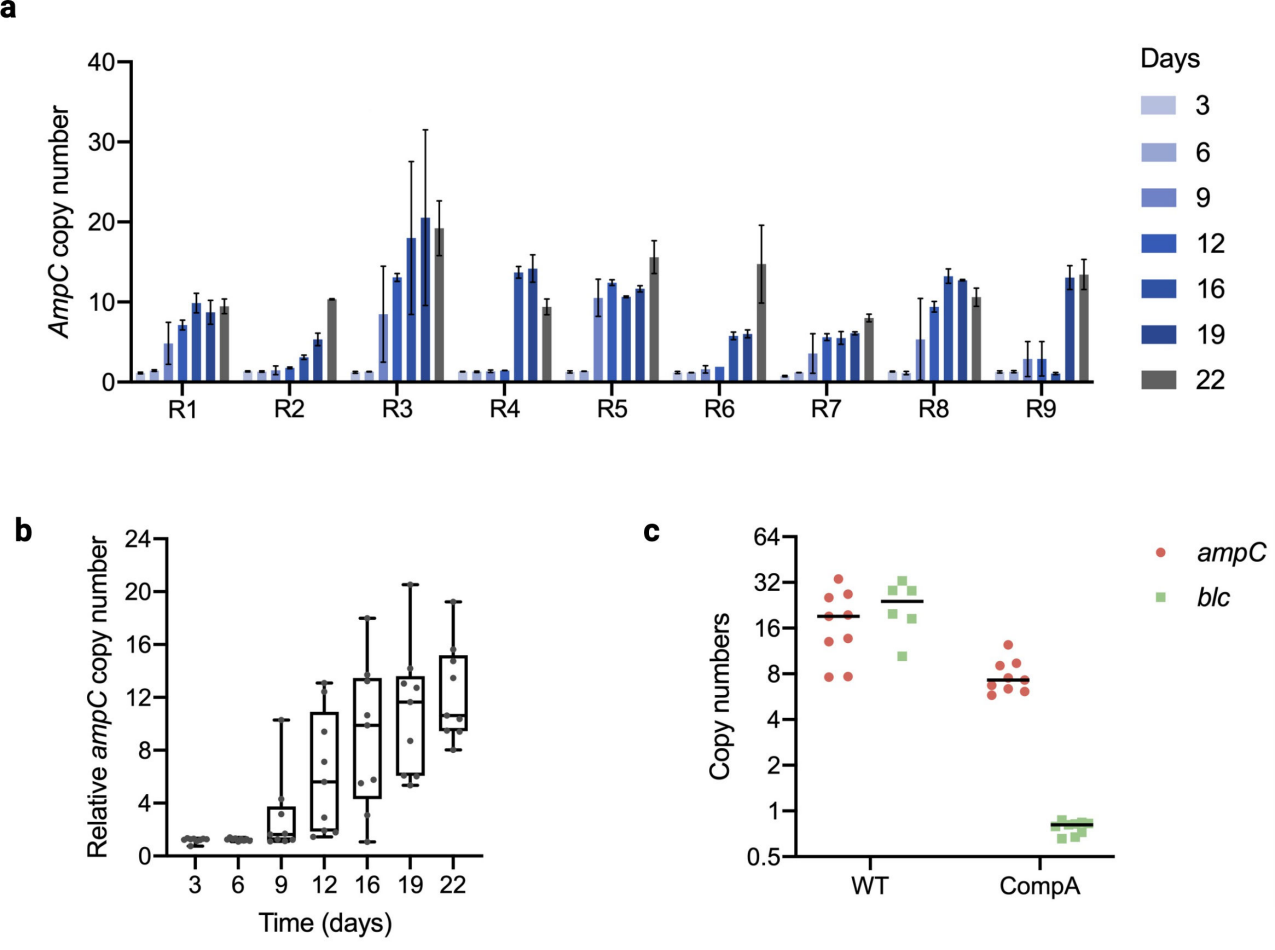
*ampC* copy number during the evolution. (**a**) *ampC* copy number at the indicated time points for each of the nine WT replicates indicated as R1-9. Time points are marked by different colors. (**b**) Distribution of *ampC* copy number at each time point in the WT replicates. The box indicates the lower 25% and higher 75%. The lowest and highest data points are indicated by the thin lines and the median by the horizontal line in the box, respectively. (**c**) Copy number of *ampC* (red) and *blc* (green) in WT and CompA after the evolution.

The *ampC* gene coding for a beta-lactamase can be amplified when the cells are exposed to stepwise increasing non-lethal levels of amoxicillin (16). In order to determine the moment that this amplification takes place, the copy number of the *ampC* gene in the genome of the evolving strains was measured at 3-day intervals using qPCR in all nine WT replicates (Fig. 2a). In the first 6 days, the *ampC* copy number did not increase in any of the replicates. The first amplifications were observed on day 9 (Fig. 2b). After that, in only one replicate did the copy number seem to increase gradually. In all other replicates, an initial jump of a factor eight was observed. The median time point for amplification was around day 12; the last single copies were seen on day 16; and at days 19 and 22, all copy numbers ranged from 8 to 19.

**Fig 3:**
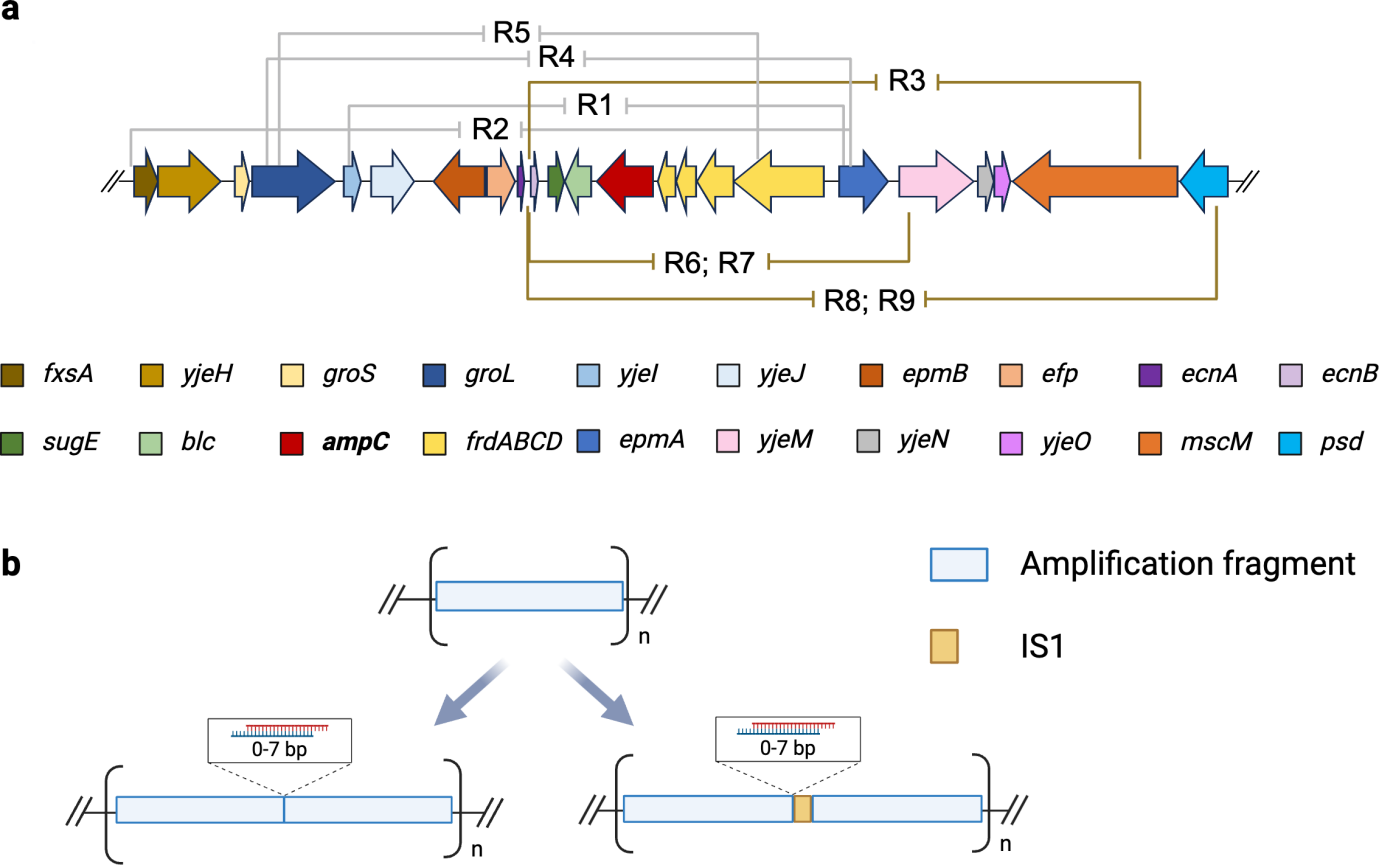
Two types of the *ampC* amplification junctions observed in the parallel evolved WT. (**a**) Various amplification fragments in evolved WT for the indicated replicates. Arrows with different colors represent genes contained in the fragment. Brown and gray lines indicate amplification contigs with and without IS*1* in their junctions, respectively. (**b**) Junction types between amplification fragments with and without IS*1*.

The *ampC* copy number was measured in resistant CompA after the 22-day resistance evolution. The copy number of *blc*, which is the gene next to *ampC* in the WT, was also assessed. The *ampC* amplification occurred in CompA, as well (Fig. 2c). Combined with the observation that WT and CompA build up similar resistance that is higher than the acquired resistance of ∆*ampC*, this confirms the essential role of *ampC* amplification to the development of resistance. In order to determine the size and composition of the amplified DNA fragment, whole genome sequencing was performed on all replicates of the WT, three replicates of ∆*ampC*, and three replicates of CompA at the end of the resistance evolution. Seven different amplified fragments were found, of which five were unique, and two fragments were observed twice (Fig. 3a). The length of the amplified region ranged from 7.5 to 13.4 kb. Five fragments shared the same terminal located within the *ecnB* promoter or reading frame, indicating that a preferred target exists for initiation of the amplification. One terminal sequence located in *epmA* is shared by three fragments, each with a different terminal on the other site. All amplified fragments contain the *sugE*, *blc*, and *ampC* genes. *sugE* codes for efflux transporters of the small multidrug resistance protein family (23), while *blc* codes for an outer membrane lipoprotein (24).

**Fig 4:**
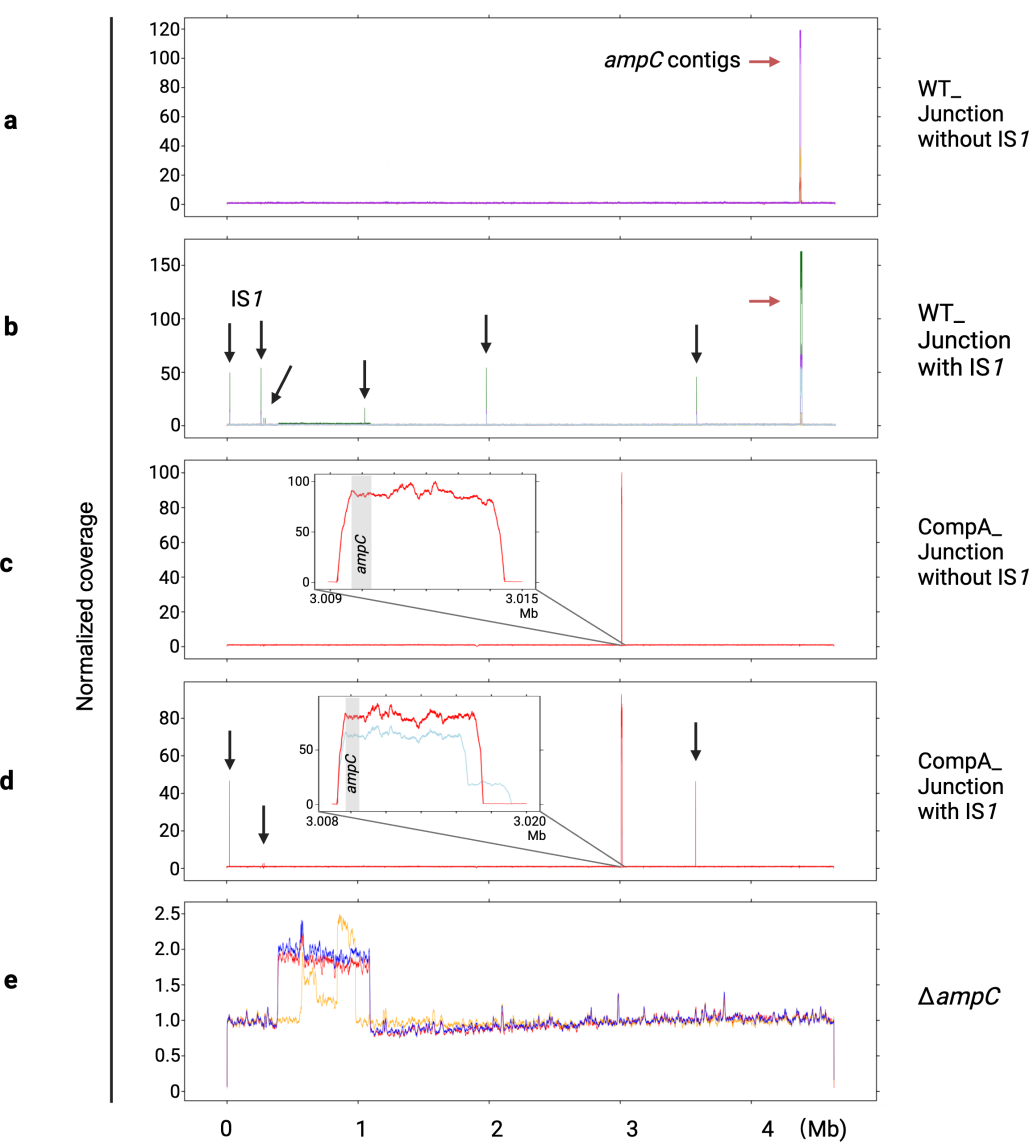
Normalized DNA copy number across the genome in whole genome sequencing. (**a** and **b**) Copy numbers of the evolved WT without and with IS*1* in the amplification junction, respectively. Note that the *Y*-axes are not identical in the five plots. *AmpC* contigs are pointed out with red arrows. IS*1* is pointed out with black arrows. (**c** and **d**) The evolved CompA without and with IS*1* in the amplification junction. The zoom-in plot shows the region around the *ampC* gene (gray box). (**e**) Evolved ∆*ampC* strains. Different colors represent different parallel evolution replicates.

We tested whether the amplification fragments are tandemly connected by PCR, designing the forward primer to bind to the end sequence and the reverse primer to bind to the start sequence of the amplified fragments. The junction between two copies of amplified contigs was confirmed by Sangar sequencing. Two types of junctions were found (Fig. 3b). In four of nine WT replicates, two amplification contigs were directly connected, with 0 to 7 bp overlapping base pairs. In the other five, an IS*1* transposon of 768 bp was inserted in the junctions with also a 0 to 7 bp homology base pair between one amplification fragment and the IS*1* element. Interestingly, all replicates with IS*1* inserted into the amplification junction have one of their terminals located in the *ecnB* promoter or reading frame. There are eight copies of transposon IS*1* in the naive WT genome. The copy number of IS*1* increased in the evolved WT strains that had IS*1* in their amplification junctions (Fig. 4b), whereas that of IS*1* was unchanged in those strains without IS*1* in the junction (Fig. 4a). However, there is no difference in the phenotype of the resistant strains with or without IS*1* in the amplified fragment.

The length of the amplified region in evolved CompA ranged from 6.5 to 11.2 kb. The same amplification junction types were observed in evolved CompA. Two in three sequenced CompA replicates had IS*1* insertions in the amplification junction, while the other one did not. The copy number of IS1, similar to the evolved WT, increased in the evolved CompA strains with IS*1* insertion in the amplification junction (Fig. 4d) and remained unchanged in the one without IS*1* in the junction (Fig. 4c).

In evolved ∆*ampC*, there was no amplification near the original *ampC* position, indicating that the other genes in the amplification fragment of evolved WT contribute much less or not at all to amoxicillin resistance (Fig. 4e). Instead, a large chromosomal duplication or triplication occurred in similar positions between 0.388 and 1.391 Mb on the chromosomal map. Genes in this region that may contribute to amoxicillin resistance include *acrA*, *acrB*, *ompF*, sulA, and *ftsZ*.

### DNA mutations in the evolved WT and ∆*ampC*

**Fig 5:**
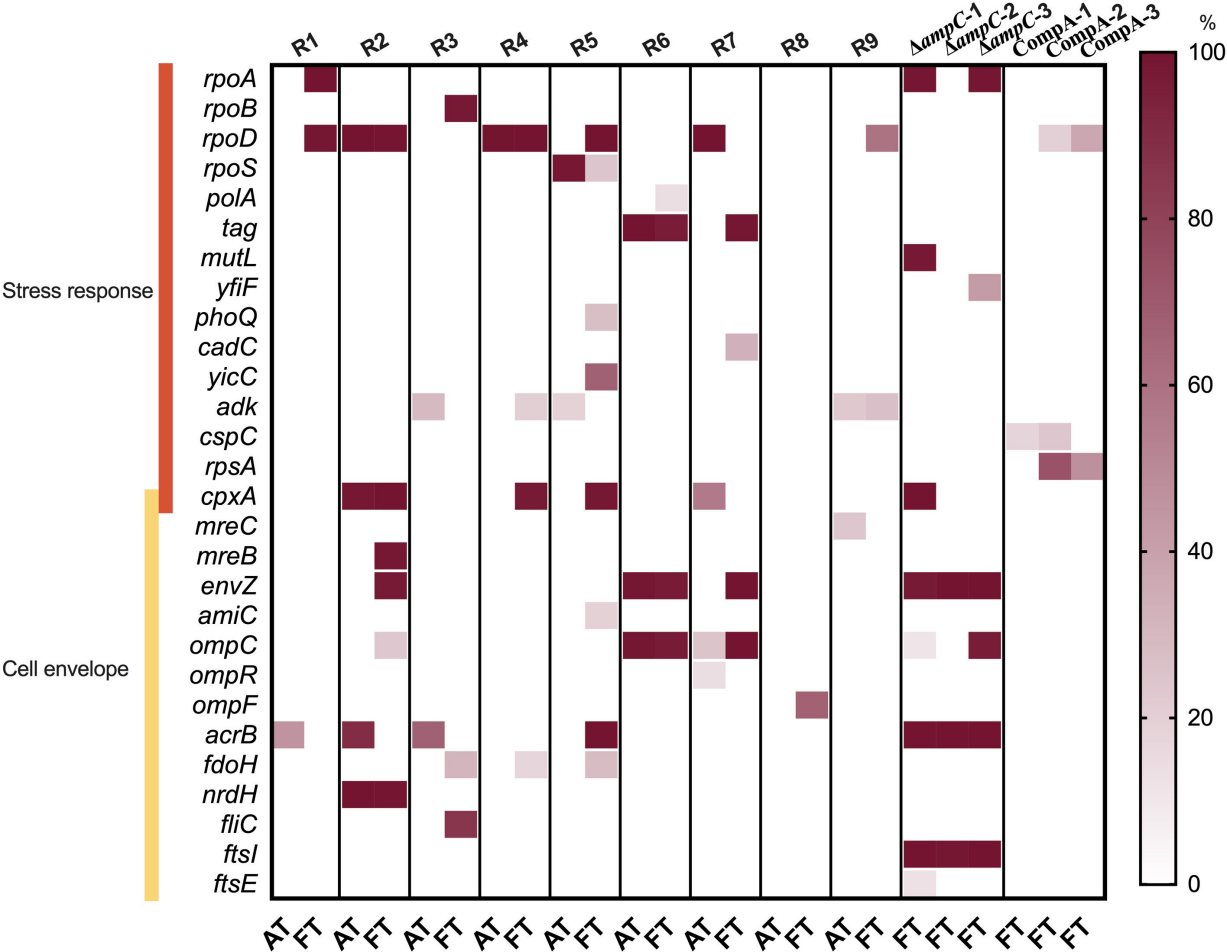
SNPs associated with *ampC* amplification in amoxicillin evolution. The color intensity of the squares indicates the frequency in the replicates of mutations in the genes named on the left side. The time points are the moment of first *ampC* amplification (AT) and the end of the experiment inducing resistance (FT). Note that not one gene is mutated in all WT or CompA replicates, although three are in the three ∆*ampC* replicates. R1 to R9 are the evolved replicates of WT, while ∆*ampC*-1 to ∆*ampC*-3 and CompA-1 to CompA-3 are the three sequenced evolved strains of ∆*ampC* and CompA, respectively.

To find which genes are mutated in association with the amplification of *ampC*, whole genome sequencing was performed on DNA isolated at the moment of *ampC* amplification (AT) and the end of the evolution experiment (FT) in WT strains, as well as the ∆*ampC* and CompA strains at FT. Mutations were identified through alignment to the *E. coli* genome downloaded from the National Center for Biotechnology Information, eliminating those also present in the naïve controls and those observed at frequencies below 10%. Excepting mutations related to *ampC*, a total of 99 DNA mutations (84 single-nucleotide polymorphisms, five deletions, and 10 insertions) were identified in all evolved WT replicates (Tables S1 and S2). Of these, 32 of single-nucleotide polymorphisms (SNPs) were outside of reading frames. In the evolved ∆*ampC* strains, we identified 97 DNA mutations with the same analysis workflow (96 SNPs and one deletion), 55 of them are outside of gene reading frames (Tables S2 and S3). The genes of interest are divided into two functional groups, stress response and cell envelope, and presented in a heatmap indicating the frequencies (Fig. 5).

There is no common mutation shared by all sequenced resistant strains. In the evolved WT, several SNPs associated with stress response are identified in genes coding for sigma factors (*rpoA*, *rpoB*, *rpoD*, and *rpoS*). Most SNPs are observed in *rpoD* that contains mutations in six out of nine replicates. The majority of these mutations show an increasing or sustained frequency from the time point at which the first amplification was observed to the end of the evolution experiment. In one of the replicates, the mutations in *rpoD* appear to outcompete those in *rpoS*, as evidenced by a decreased mutation frequency in *rpoS* and a simultaneous increase in *rpoD*.

The evolved ∆*ampC* strains tend to exhibit mostly SNPs associated with membrane-related processes. All ∆*ampC* replicates contained SNPs in *ftsI*/*acrB*/*envZ*. The inhibition of FtsI activity by binding of beta-lactam antibiotics is lethal, as this is an essential cell division protein (25). Although the gain-of-function mutation in *ftsI* is well known for amoxicillin resistance (26), this mutation did not occur in evolved WT strains. The proportion of mutations located in *envZ*/*ompC*/*acrB* is higher than in the WT, suggesting an increasing role of other resistance pathways when *ampC* is absent. The SNPs in *rpoD*, which had a high frequency in evolved WT, were not observed in ∆*ampC*. However, this SNP was observed again when *ampC* was inserted in another location in the chromosome in CompA. This suggests that this mutation correlates with resistance mechanisms based on *ampC* overexpression.

### Relationship between *ampC* mRNA level and *ampC* copy number

**Fig 6:**
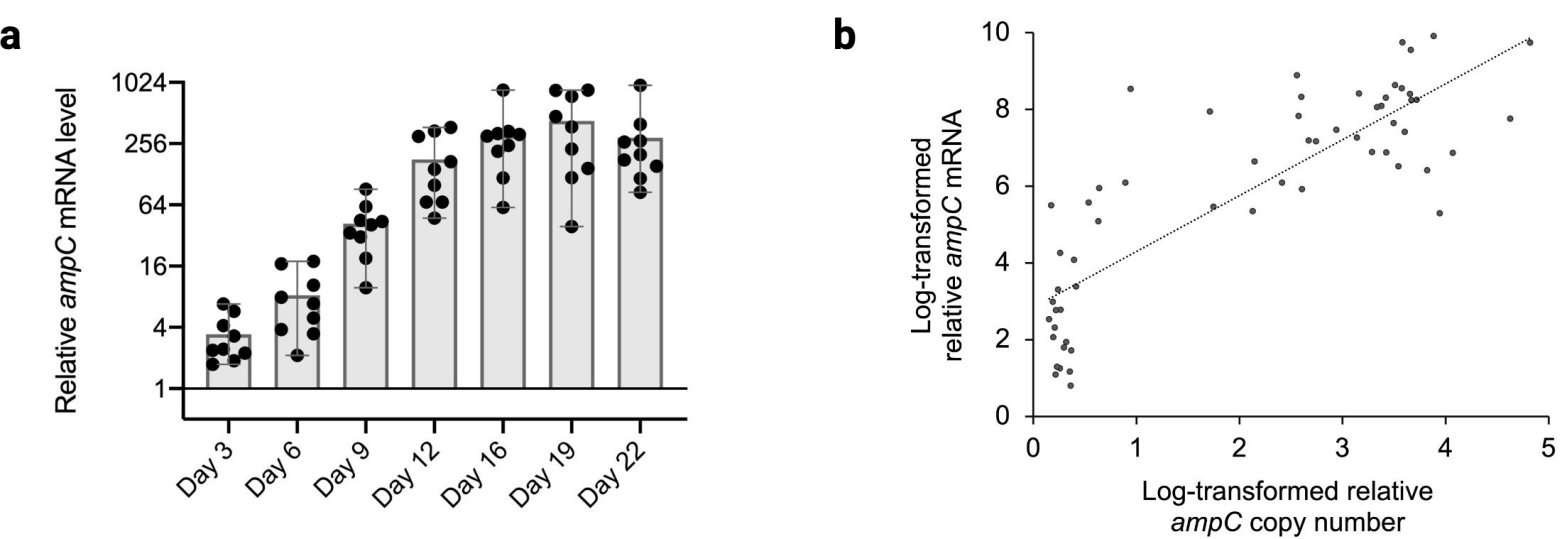
*ampC* transcription level in amoxicillin evolution. (**a**) Fold change in the *ampC* expression compared to the native WT. Note that the *Y*-axis is in log scale. One is the average of the WT replicates. Each point represents the mean of three technical replicates. Horizontal spacing is solely to avoid printing points on top of each other. (**b**) Relationship between the *ampC* copy number and the *ampC* mRNA level. The log-transformed relative copy number and mRNA were fitted into a linear trendline.

To determine the consequences of the observed *ampC* gene copy number increase, *ampC* mRNA levels were measured at the same time points that the *ampC* copy number was ascertained (Fig. 6a). Even though there was no gene amplification within the first 6 days of the evolution experiments, a continuous increase of *ampC* mRNA levels was evident, indicating that other factors also enhance *ampC* transcription ahead of *ampC* amplification. During induction of resistance, *ampC* mRNA levels significantly increased in all replicates, ranging from 85- to 961-fold change compared to the WT.

In order to establish the relationship between the *ampC* copy number and mRNA level, the *ampC* mRNA level and copy number fold change were transformed by log_2_ and fitted in linear function (Fig. 6b). The *ampC* mRNA level and copy number show a positive correlation. However, the linear formula does not fit well. This suggests that other factors, in addition to the *ampC* copy number, also have considerable impact on *ampC* gene transcription.

### Trajectory of mutations related to *ampC*

Instigated by the evidence above, we further explored how the other mutations affect *ampC* transcription. Besides gene dosage, promoter activity is another crucial factor influencing gene transcriptional levels. Therefore, the *ampC* promoter region was sequenced at eight different time points during the evolution experiments (Table S4). *AmpC* promoter mutations were observed as early as day 2, affecting the −10 box of the promoter. As evolution progressed, more mutations emerged. However, not all of them were retained until the final days. The first mutation in the *ampC* promoter occurred earlier than *ampC* amplification. Combining this information with the observation that *ampC* mRNA levels increase by about 10-fold prior to *ampC* gene amplification suggests that the mutated *ampC* promoter is responsible for the initial increase in *ampC* transcription.

**Fig 7:**
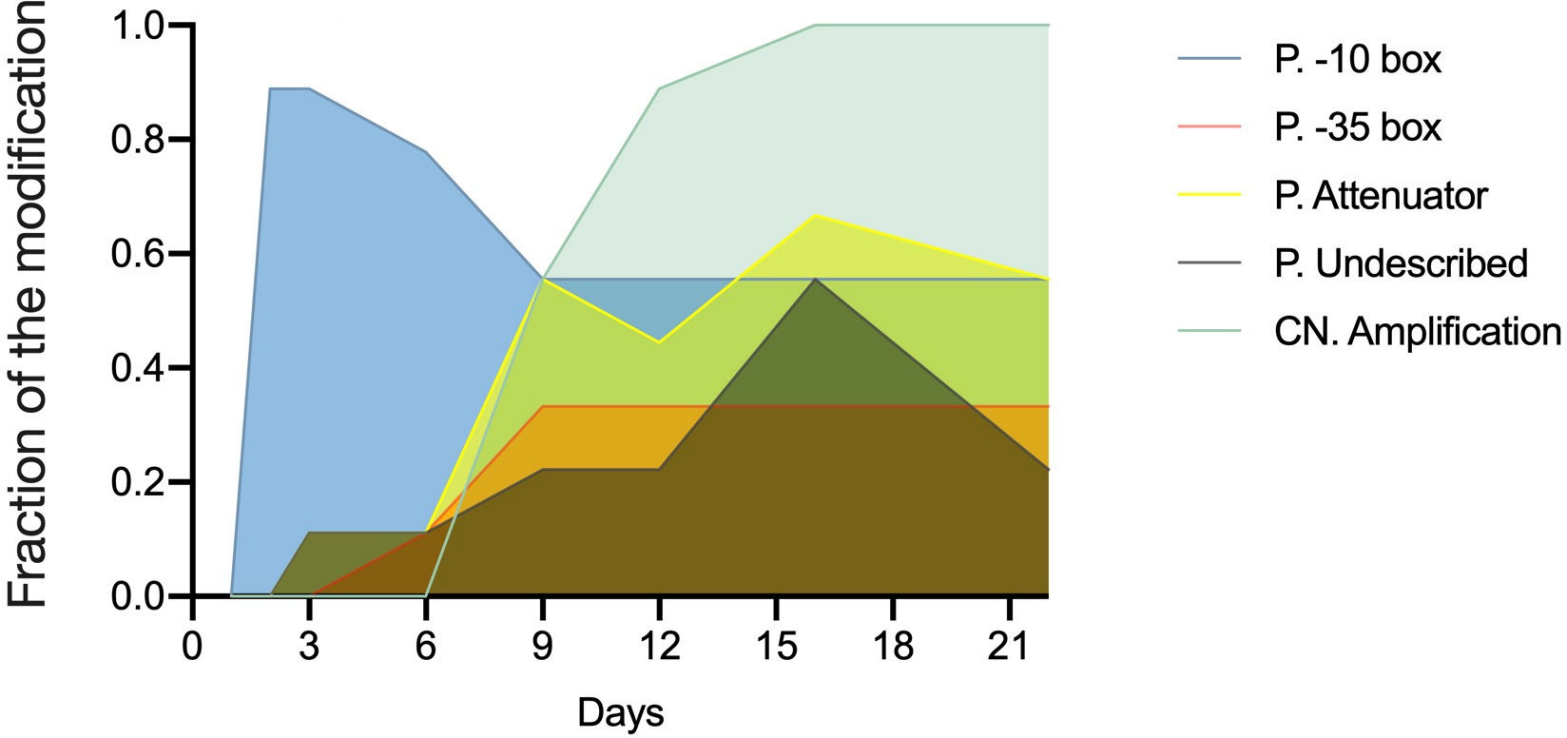
Trajectory of mutations in the *ampC* promoter and copy number change during resistance evolution in the WT. Mutation frequency was calculated over the nine evolved replicates. P. represents the *ampC* promoter. CN. represents the *ampC* copy number. Mutations throughout the remainder of the chromosome are not included. Different elements in the promoter and the copy number variation are represented by different colors.

The mutations in the *ampC* promoter occurred in three main elements: the −10 box, the −35 box, and the attenuator. In the nine replicates, the mutations in the −10 and −35 boxes were conserved, −11 G > A in the −10 box, and −32 A > T in the −35 box, but not those in the attenuator. In the attenuator area, five different mutations were found in different replicates at the end of the resistance evolution. Additionally, there were a few mutations in other sites that have not been reported before in the *ampC* promoter. To uncover a possible influence of changes in the *ampC* promoter region on the *ampC* copy number, the trajectory of mutations in the *ampC* promoter region was documented (Fig. 7). Mutations in the −10 box already occurred by day 2. However, the frequency of this mutation decreased after day 3, accompanied by an increase in the frequency of mutations in the −35 box. After the *ampC* copy number started to increase, mutations in the −10 and −35 boxes did not show systematic changes. Mutations in the attenuator and undescribed area were more unpredictable, with their frequency continuously changing throughout the entire evolution process.

## DISCUSSION

Strains with higher AmpC production have a selective advantage in amoxicillin resistance development. The overexpression of the AmpC enzyme can be achieved by the enhancement of *ampC* gene transcription by increasing the *ampC* promoter strength and the gene copy number. When exposed to increasing concentrations of amoxicillin, *E. coli* gained resistance by increasing the transcription of *ampC* by a factor exceeding 100 (9). This study reports the time course of the amplification of a chromosomal fragment containing *ampC* and the genetic events accompanying it. The considerable delay before the first amplification events observed in this study indicates that the mutated *ampC* promoter causes the initial increase of AmpC activity and amoxicillin resistance. Therefore, the process of amplification is not gradual. Instead, the initial jump in copy number seems to be around 8-fold and occurs after at least 6 days of exposure to increasing amoxicillin levels.

The mobile genetic elements could drive the antibiotic resistance genes’ copy number increase (15). IS*26* mediates the amplification of *bla_KPC-2_* in an *E. coli* clinical strain under sublethal meropenem or tobramycin exposure (27). The chromosomal DNA fragment with *ampC* isolated from *E. coli* made amoxicillin resistant by *de novo* evolution contains the IS*1* transposon, which was introduced at the connection of the start and end of the fragments. This fragment can transfer from the amoxicillin-evolved *E. coli* functioning as donor to susceptible *E. coli* receptor cells (16). In *Proteus mirabilia*, amplification involving IS*1* is based on homology recombination identifying two IS*1* copies as homology regions for initial recombination, followed by tandem duplication of the region between IS*1* elements (28). In this IS*1*-mediated amplification, the frequency of the initial duplication is 150-fold lower than that of the following amplification to higher copy numbers (29). If this is the same in *E. coli*, that would explain the pattern of *ampC* amplification observed here. All replicates with IS*1* inserted in the junction of two amplification fragments had one of their amplifications flanking within the *ecnB* reading frame or promoter. The extremities of the IS*1* sequence in particular are crucial for cointegration (30). The *ecnB* promoter and the tail of IS*1* share the same 7 bp sequence. This implies that P*ecnB* contains a region homologous to part of IS*1*, suggesting a potential function of this sequence as the required homologous region for the recombination event, recruiting IS*1* transposon and leading to amplification. Between the replicates with and without IS*1* inserted in the amplification junction, there is no phenotypical difference. Combined with the observation that frequency of these two junctions were the same, it seems that both mechanisms, IS*1* involved or not, offer equal evolutionary advantages.

The absence of amplification in the original position of *ampC* in evolved ∆*ampC* strains indicates that none of the other genes in the fragment confer enough resistance advantage for its amplification. It also rules out the already unlikely possibility that the cell would be aware of the location of *ampC* in the genome and amplify the region around it when exposed to amoxicillin. Instead, a large region containing more than 1,000 genes was duplicated or triplicated. A similar amplification was also observed in tetracycline-evolved *E. coli* (31), indicating that the driving factor may be the presence of multi-drug resistance genes within the amplified region.

Gene expression involves the coordination of multiple dynamic events subject to multi-level regulation (32). The positive relationship between AmpC activity and the MIC, *ampC* copy number, and mRNA levels suggests that according to the hierarchical control analysis (33), the genetic control component fully determines levels of expression, which in turn control AmpC activity. Hence, the *ampC* copy number exerts considerable influence over its expression, but that it is not the only factor. The mutations in the promoter areas are crucial in the initial stages of resistance development.

The comparison of gene mutations during evolution in WT and ∆*ampC* suggests that the deletion of *ampC* not only reduces the ability to acquire resistance but also alters the evolutionary trajectory in *E. coli*. The ability to adapt and the alteration of mutations pattern were reversed by the complementation of *ampC* gene at another location in the genome. Point mutations in several genes exhibited higher frequency in evolved ∆*ampC* strains compared with WT, suggesting that additional mutations were needed to compensate for the missing *ampC* gene. The mutations in gene *ftsI* and *acrB* occur in all ∆*ampC* strains. These genes are known to confer amoxicillin resistance based on pathways associated with target alternation (34) and efflux pumps (35), respectively. In contrast, in the WT, the mutation in *acrB* disappeared from the time point of the first amplification observed to the end of the evolution experiment in three replicates, and the mutation in *ftsI* was not observed. This suggests that these mutations may cause higher fitness costs than *ampC* amplification. Besides, mutations in *envZ* may confer resistance by decreasing drug influx through the membrane porins OmpC/OmpF (20, 36). Moreover, these mutations have been shown to increase carbapenem resistance in *ompCF*-deleted backgrounds (37). Mutations in the *envZ* gene occur in all evolved ∆*ampC* strains and also accumulate in several evolved WT strains, indicating that they synergize with AmpC.

Several DNA mutations present in the WT after resistance evolution were not observed in evolved ∆*ampC*. The mutations in *rpoD* accumulated in most evolved WT. However, this did not occur in evolved ∆*ampC*, indicating that *rpoD* may be important for *ampC* transcription enhancement. RNA polymerase coded by sigma factor 70 (σ^70^) gene *rpoD* is essential for gene transcription (38). RpoD connects to both the −10 and −35 regions in the promoter to initiate transcription (39). A mutation at the same position in *rpoD* (Asp445Glu) was recently reported in cefotaxime- and ampicillin-resistant *E. coli* (26, 40). Mutations in the *rpoD* gene in our study changed amino acids 445 (Asp445Ala, Asp445Val), 447 (Ala447Pro), and 570 (Asp570Gly) in several replicates. These sites are in the conserved regions 2.4 and 4.2 of the σ^70^ subunit, which connect to the −10 and −35 motifs within the promoter, respectively (41). As mutations in *rpoD* occur later than *ampC* duplication and mutations in −10 and −35 boxes of the *ampC* promoter, the σ^70^ mutants potentially enhance binding affinity between the RNA polymerase and gene promoter, thereby improving the utilization of high gene dosage.

## MATERIALS AND METHODS

### Strains, culture conditions and adaptive evolution

Minimal medium, pH 6.9, supplemented with 55 mM glucose (42) was used for the amoxicillin resistance evolution of the *E. coli* MG1655 (WT) and ∆*ampC* (JW4111 from NBRP *E. coli* Strain Collection, Japan; kanamycin resistance was removed). The Luria–Bertani ( medium was used for the evolution of CompA, as well as for the WT control for that experiment, since this strain does not grow in minimal medium. Strains were cultured at 37°C, with shaking at 200 rpm. Stock solutions of amoxicillin (10 mg/mL) were dissolved in 50 mM HCl solution and filter-sterilized. The evolved strains were stored with 30% glycerol at −80°C.

The resistance evolution experiments were performed as described before (43). Nine replicate evolutions of WT, six of ∆*ampC*, and nine of CompA were performed to develop amoxicillin resistance. One culture of each strain passaged in a drug-free medium was used as the control. The starting amoxicillin concentration was 1 µg/mL for all strains. The initial OD_600_ was 0.1. After 24 h grown in 10 mL tube with 5 mL medium, if the OD_600_ of the culture in higher concentration was higher than 70% that in lower concentration, the antibiotic concentration was doubled in the subsequent incubation, otherwise using the same antibiotic concentration.(44) The evolution experiments standardized at 22 days, as after that time maximum resistance was reached, and compensatory mutations do not yet occur.

### MIC measurement

Because at least seven time points are needed for a linear or quadratic regression model (45), during the evolution process, the minimal inhibitory concentration (MIC) values were measured every 3 to 4 days to obtain seven time points at the end of the 22-day evolution experiment. The MIC measurement was performed in 96-well plates in plate readers (Thermo Scientiﬁc Multiskan FC with SkanIt software), as described before (46). Amoxicillin concentrations ranged from 2 to 2,048 µg/mL, increasing by a factor of 2 at each step. The initial OD_600_ was 0.05. Plates were incubated at 37°C for 24 h, with shaking and OD_595_ measurements conducted every 10 min. The MIC was deﬁned as the lowest amoxicillin concentration that reduced the growth to OD_595_ less than 0.2 after 24 h incubation.

### AmpC activity measurement

The stored strains were grown in Evans medium overnight from storage tubes kept at −80°C and diluted 1:100 with fresh Evans medium. Cultures were harvested at late-log phase and washed in 1 M PBS 7.0 buffer. Cells were diluted to OD_600_ 2 with PBS. Next, 50 µL cell suspension was mixed with 50 µL of 10 µg/mL nitrocefin and incubated at 37°C in a plate reader with pulse shaking. The OD_492_ was measured every minute for 10 h. The fold change of the activity was calculated by dividing the activity of evolved strains by that of naive WT.

### Quantitative PCR

Genomic DNA in 1 mL evolved strain culture was extracted with the DNeasy Blood and Tissue Kit (QIAGEN GmbH, Germany) for copy number measurements and whole genome sequencing. RNA was isolated using the RNeasy Protect Bacteria Kit (QIAGEN GmbH, Germany), and reverse transcription was carried out with the iScript cDNA Synthesis Kit (Bio-Rad, USA).

TaqMan Universal PCR Mix (Thermo Fisher, UK) was used for quantitative PCR (qPCR) performed with the Applied Biosystems 7300 Real-Time PCR System (Applied Biosystems). Primers and probes for qPCR (Table S5) were obtained from Integrated DNA Technologies (Leuven, Belgium), and 6-FAM and TAMRA were used as dye and quencher of the probe, respectively. A sample of naive WT was used as reference. The cDNA or genomic DNA was diluted to the same concentration (10 ng/µL). Cycle threshold (Ct) values were determined by automated threshold analysis using ABI Prism 1.0 software. Gene copy number or gene relative production was determined using the −ΔΔCt method using GADPH as the reference gene.

### Sequencing of the *ampC* promoter

The *ampC* promoter was amplified by PrimeSTAR Max DNA polymerase (TaKara, China) using isolated genomic DNA as template and F-AmpC Prom and R-AmpC Prom (Table S5) as primers. The PCR product was purified using the MSB Spin PCRapace Kit (Invitek Molecular GmbH, Germany) and sequenced by Sangar (Macrogen Europe). The result was analyzed through SnapGene. Only the highest signal of mutations at each site was recorded.

### Whole genome sequencing

Whole-genome sequencing was conducted by utilizing next-generation sequencing Illumina (NextSeq 550 System) following the established protocol (43). The NEBNext Ultra II FS DNA Library Prep Kit for Illumina (New England BioLabs, USA) and NEBNext Multiplex Oligos for Illumina (96 Unique Dual Index Primer Pairs; New England BioLabs, USA) were used for creating a genomic DNA library. After removing the adapter using Cutadapt (47), the raw data were trimmed (48) and deduplicated. The bam files were then aligned to references (NC000913 for WT, CP009273 for ∆*ampC* and CompA) with bowtie2 (49). SAMtools depth was used for depths and coverage confirmation. Freebayes (50) and Lofreq (51) were used for allele frequency calculation and variant calling, respectively. SnpEff (52) was used for variant annotation. The point mutations with allele frequency lower than 0.1 and those also occurring in the drug-free cultured control were removed.

The structure variation was further confirmed through Breseq (53) and cn.mops (54). The trimmed fastq file was used for Breseq for transposition identification. The range of the amplification fragment was determined from the output of cn.mops. These functions were conducted with bam file without deduplication following Zhou et al. (55).

### Statistical analysis

The Wilcoxon signed-rank test was used to compare the resistance adaptation rate and MIC differences between various strains (56). Initially, the median was calculated with all replicates at each time point for each strain. The *P* value between data sets was then calculated using the function wilcon_test in R. Student’s *t*-test was used for the *ampC* copy number comparison between resistant WT and CompA in GraphPad Prism v8.3.1.

## SUPPLEMENTAL MATERIAL

Supplemental tables mbio.02982-24-s0001.pdf Tables S1 to S5.

ASM does not own the copyrights to Supplemental Material that may be linked to, or accessed through, an article. The authors have granted ASM a non-exclusive, world-wide license to publish the Supplemental Material files. Please contact the corresponding author directly for reuse.
