# Supplementary material for: Progression of ampC amplification during de novo amoxicillin resistance development in E. coli
Source: mBio. 2024 Dec 20;16(2):e02982-24. doi: 10.1128/mbio.02982-24 (PMC11796351; doi:10.1128/mbio.02982-24)
Supplement: Supplemental tables — Tables S1 to S5. [file mbio.02982-24-s0001.pdf]

Table S1 Single-nucleotide polymorphisms in evolved WT

Position	Annotation	Referece base	Altered base	Altered amino acid	Frequency																	
					R1_FT	R1_AT	R1_FT	R1_AT	R1_FT	R1_AT	R1_FT	R1_AT	R1_FT	R1_AT	R1_FT	R1_AT	R1_FT	R1_AT	R1_FT	R1_AT	R1_FT	R1_AT
1527188	*from_yncH_ to_ydcD*	T	C	-	0.99	1.00	1.00	1.00	0.98	0.96	1.00	1.00	1.00	0.99	0.95	1.00	0.99	1.00	-	-	-	-
3214380	*rpoD*	A	C	Asp445Ala	0.99	-	-	-	-	-	1.00	1.00	-	-	-	-	-	-	-	-	-	-
3214755	*rpoD*	A	G	Asp570Gly	0.93	-	-	-	-	-	-	-	-	-	-	-	-	-	-	-	-	-
1875676	*nimT*	A	C	Thr2Pro	0.88	-	-	-	-	-	-	-	-	-	-	-	-	-	-	-	-	-
3998195	*uvrD*	T	G	His72fs	0.38	-	-	-	-	-	-	-	-	-	-	-	-	-	-	-	-	-
302463	*paoA*	C	G	Ala37Ala	0.34	-	-	-	-	-	0.29	0.33	-	-	-	-	-	0.38	0.36	-	-	0.33
2718321	*yfiF*	G	C	Arg70Gly	0.32	-	-	-	-	-	-	-	-	-	-	-	-	-	-	-	-	-
2074685	*from_flu_ to_yeeR*	G	C	-	0.31	-	0.34	-	-	-	0.33	-	-	-	0.35	-	-	-	-	-	-	-
707976	*from_glnS_ to_chiZ*	T	G	-	0.19	0.28	-	0.27	0.22	0.33	-	-	-	-	-	0.25	0.27	0.20	-	0.20	-	0.28
1381291	*from_ycjU_ to_ompG*	A	T	-	0.17	-	-	-	-	-	-	-	-	-	-	-	-	-	-	-	-	-
2736931	*from_bamD_ to_raiA*	A	T	-	0.13	-	-	-	-	-	-	-	-	-	-	-	-	-	-	-	-	-
3194593	*from_yqiK_ to_sibD*	A	G	-	0.11	-	-	-	-	-	-	-	-	-	-	-	-	-	-	-	-	-
3440212	*rpoA*	T	C	Glu273Gly	1.00	-	-	-	-	-	-	-	-	-	-	-	-	-	-	-	-	-
2800855	*nrdH*	G	A	Ala45Thr	-	-	0.99	1.00	-	-	-	-	-	-	-	-	-	-	-	-	-	-
3214380	*rpoD*	A	T	Asp445Val	-	-	0.99	1.00	-	-	-	-	-	-	-	-	-	1.00	-	-	-	-
3400123	*mreB*	A	C	Leu322Arg	-	-	0.98	-	-	-	-	-	-	-	-	-	-	-	-	-	-	-
3535354	*envZ*	A	C	Ile172Arg	-	-	0.97	-	-	-	-	-	-	-	-	-	-	-	-	-	-	-
4092667	*frvA*	A	G	Ser53Pro	-	-	0.91	-	-	-	-	-	-	-	-	-	-	-	-	-	-	-
4092664	*frvA*	T	C	Thr54Ala	-	-	0.86	-	-	-	-	-	-	-	-	-	-	-	-	-	-	-
4092660	*frvA*	C	CGCGT	Gly55fs	-	-	0.67	-	-	-	-	-	-	-	-	-	-	-	-	-	-	-
4095852	*rhaA*	T	C	Asp44Gly	-	-	0.67	-	-	-	-	-	-	-	-	-	-	-	-	-	-	-
3233718	*from_fadH_ to_higA*	G	C	-	-	-	0.42	0.37	-	0.39	-	-	0.48	-	-	-	-	-	0.57	-	0.36	-
3491701	*from_yhfG_ to_ppiA*	C	G	-	-	-	0.38	-	0.42	-	0.28	-	-	0.28	0.29	-	-	-	-	0.33	0.32	-
2312798	*from_ompC_ to_micF*	C	CAGGTG	-	-	-	0.23	-	-	-	-	-	-	-	-	-	-	-	-	-	-	-
2204829	*yehL*	T	G	Gly78Gly	-	-	0.18	-	-	-	-	-	-	-	-	-	-	-	-	-	-	-
4094134	*rhaD*	C	T	Asp47Asn	-	-	1.00	-	-	-	-	-	-	-	-	-	-	-	-	-	-	-
4184040	*rpoB*	GGTCTTTACTCGC	G	Val933_Arg936del	-	-	-	-	0.98	-	-	-	-	-	-	-	-	-	-	-	-	-
1799271	*from_pheM_ to_rplT*	T	C	-	-	-	-	-	0.97	0.99	-	-	-	-	-	-	-	-	-	-	-	-
720884	*from_speFL_ to_kdpE*	A	G	-	-	-	-	-	0.92	-	-	-	-	-	-	-	-	-	-	-	-	-
2003387	*fliC*	C	A	Val74Phe	-	-	-	-	0.85	-	-	-	-	-	-	-	-	-	-	-	-	-
4531697	*from_sgcX_ to_yjhP*	T	C	-	-	-	-	-	0.56	-	-	-	-	-	-	-	-	-	-	-	-	-
4531698	*from_sgcX_ to_yjhP*	A	C	-	-	-	-	-	0.56	-	-	-	-	-	-	-	-	-	-	-	-	-
4082690	*fdoH*	G	C	Arg24Gly	-	-	-	-	0.31	-	-	-	-	-	-	-	-	-	-	-	-	-

3623441	*yhhH*	A	ACC	Arg15fs	-	-	-	-	0.29	-	-	-	-	-	-	-	-	-	-	-	-	-
151734	*yadL*	A	C	Val166Val	-	-	-	-	0.26	-	-	-	-	-	-	-	-	-	-	-	-	-
2725790	*from_kgtP_ to_yfiS*	T	G	-	-	-	-	-	0.25	-	-	-	-	-	-	-	-	-	-	-	-	-
2725794	*from_kgtP_ to_yfiS*	G	T	-	-	-	-	-	0.23	-	-	-	-	-	-	-	-	-	-	-	-	-
3136631	*from_pitB_ to_gss*	T	G	-	-	-	-	-	0.11	-	-	-	-	-	-	-	-	-	-	-	-	-
4641647	*after_yjtD*	T	TTTTTCAGC	-	-	-	-	-	1.00	-	-	-	-	1.00	-	1.00	-	-	1.00	-	-	-
4104875	*cpxA*	T	G	Gln34Pro	-	-	-	-	-	-	0.97	-	-	-	-	-	-	-	-	-	-	-
1123119	*yceB*	G	C	Ala52Gly	-	-	-	-	-	-	0.37	-	-	-	-	-	-	-	-	-	-	-
497569	*adk*	T	G	Val132Gly	-	-	-	-	-	0.29	0.20	-	-	0.19	-	-	-	-	-	-	-	0.23
4082687	*fdoH*	C	G	Asp25His	-	-	-	-	-	-	0.17	-	0.21	-	-	-	-	-	-	-	-	-
3222500	*from_ebgR_ to_ebgA*	T	G	-	-	-	-	-	-	-	0.12	-	-	-	-	-	-	-	-	-	-	-
4104924	*cpxA*	CCAGCGT	C	Thr16_Leu17del	-	-	-	-	-	-	-	-	0.98	-	-	-	-	-	-	-	-	-
3817289	*yicC*	T	C	Leu205Pro	-	-	-	-	-	-	-	-	0.61	-	-	-	-	-	-	-	-	-
289278	*from_xynR_ to_argF*	G	C	-	-	-	-	0.37	-	-	-	0.29	0.36	-	-	0.41	0.37	-	-	-	0.49	0.29
2867272	*rpoS*	T	A	Met94Leu	-	-	-	-	-	-	-	-	0.25	-	-	-	-	-	-	-	-	-
1286793	*ychS*	A	G	Lys29Glu	-	-	-	-	-	-	-	-	0.24	-	0.21	0.41	0.28	-	-	-	-	-
2725807	*from_kgtP_ to_yfiS*	G	T	-	-	-	-	-	-	-	-	-	0.21	-	-	-	-	-	-	-	-	-
1188951	*phoQ*	T	G	Thr276Pro	-	-	-	-	-	-	-	-	0.21	-	-	-	-	-	-	-	-	-
2948173	*amiC*	A	G	Phe280Leu	-	-	-	-	-	-	-	-	0.19	-	-	-	-	-	-	-	-	-
306815	*ecpC*	C	T	Glu840Lys	-	-	-	-	-	-	-	-	0.18	-	-	-	-	-	-	-	-	-
3340800	*yrbG*	T	G	Phe176Val	-	-	-	-	-	-	-	-	0.16	-	-	-	-	-	-	-	-	-
2153805	*from_ibsB_ to_mdtA*	G	A	-	-	-	-	-	-	-	-	-	0.11	-	-	0.11	-	-	-	-	-	-
2312651	*ompC*	AT	A	Asp33fs	-	-	-	-	-	-	-	-	-	-	0.97	-	1.00	-	-	-	-	-
3535126	*envZ*	G	T	Pro248Leu	-	-	-	-	-	-	-	-	-	-	0.97	-	1.00	-	-	-	-	-
3713532	*tag*	G	T	Lys147Asn	-	-	-	-	-	-	-	-	-	-	0.96	1.00	0.99	-	-	-	-	-
290630	*ykgS*	A	C	Thr41Pro	-	-	-	-	-	-	-	-	-	-	0.26	-	-	-	-	-	-	-
290632	*ykgS*	C	T	Thr41Thr	-	-	-	-	-	-	-	-	-	-	0.26	-	-	-	-	-	-	-
290627	*ykgS*	G	A	Val40Ile	-	-	-	-	-	-	-	-	-	-	0.26	-	-	-	-	-	-	-
2570475	*eutE*	A	C	Leu426Arg	-	-	-	-	-	-	-	-	-	-	0.19	-	-	-	-	-	-	-
4048768	*polA*	A	C	Lys601Asn	-	-	-	-	-	-	-	-	-	-	0.14	-	-	-	-	-	-	-
126360	*aceF*	T	C	Thr222Thr	-	-	-	-	-	-	-	-	-	-	0.11	-	-	-	-	-	-	-
4360944	*cadC*	G	C	Pro331Ala	-	-	-	-	-	-	-	-	-	-	-	-	0.33	-	-	-	-	-
2521008	*from_valU_ to_valX*	C	T	-	-	-	-	-	-	-	-	-	-	-	-	-	-	-	0.92	-	0.67	0.90
2521007	*from_valU_ to_valX*	A	C	-	-	-	-	-	-	-	-	-	-	-	-	-	-	0.19	0.86	-	0.69	0.82
4097445	*rhaB*	T	A	Thr2Ser	-	-	-	-	-	-	-	-	-	-	-	-	-	-	0.75	0.67	-	0.85
986471	*ompF*	TC	T	Asp171fs	-	-	-	-	-	-	-	-	-	-	-	-	-	-	0.67	-	-	-
4412779	*yjfM*	T	G	Val131Val	-	0.32	-	-	-	-	-	-	-	-	-	-	-	-	0.32	-	-	-
2521010	*from_valU_ to_valX*	A	T	-	-	-	-	-	-	-	-	-	-	-	-	-	-	-	1.00	-	0.86	-
3214385	*rpoD*	G	C	Ala447Pro	-	-	-	-	-	-	-	-	-	-	-	-	-	-	-	-	0.59	-
2521013	*from_valU_ to_valX*	T	C	-	-	-	-	-	-	-	-	-	-	-	-	-	-	-	-	-	0.50	-

2521050	*from_valU_ to_valX*	T	G	-	-	-	-	-	-	-	-	-	-	-	-	-	-	-	-	-	0.45	0.44
497572	*adk*	A	T	Tyr133Phe	-	-	-	-	-	-	-	-	-	-	-	-	-	-	-	-	0.27	-
1080910	*from_putP_ to_efeO*	A	C	-	-	-	-	-	-	-	-	-	-	-	-	-	-	-	-	-	0.26	-
2521011	*from_valU_ to_valX*	C	T	-	-	-	-	-	-	-	-	-	-	-	-	-	-	-	-	-	0.25	-
2521012	*from_valU_ to_valX*	T	G	-	-	-	-	-	-	-	-	-	-	-	-	-	-	-	-	-	0.25	-
2521007	*from_valU_ to_valX*	A	G	-	-	-	-	-	-	-	-	-	-	-	-	-	-	-	-	-	0.15	0.09
2521009	*from_valU_ to_valX*	T	TAA	-	-	-	-	-	-	-	-	-	-	-	-	-	-	-	-	-	0.14	-
1397674	*uspE*	A	T	Ter317Lys	-	-	-	-	-	-	-	-	-	-	-	-	-	-	-	-	0.14	-
2817745	*from_yqaB_ to_argQ*	T	C	-	-	-	-	-	-	-	-	-	-	-	-	-	-	-	-	-	0.13	-
2818433	*from_argY_ to_argV*	C	T	-	-	-	-	-	-	-	-	-	-	-	-	-	-	-	-	-	0.12	-
2818442	*from_argY_ to_argV*	C	G	-	-	-	-	-	-	-	-	-	-	-	-	-	-	-	-	-	0.12	-

Table S2 Identified transposition and deletions in evolved strains

Position	Trans-position and deletion	Referred gene	WT_ R1_ FT	WT_ R1_ AT	WT_ R2_ FT	WT_ R2_ AT	WT_ R3_ FT	WT_ R3_ AT	WT_ R4_ FT	WT_ R4_ AT	WT_ R5_ FT	WT_ R5_ AT	WT_ R6_ FT	WT_ R6_ AT	WT_ R7_ FT	WT_ R7_ AT	WT_ R8_ FT	WT_ R8_ AT	WT_ R9_ FT	WT_ R9_ AT	Δ*ampC*_ A2_ FT
2312791	IS1 (+) +9 bp	*from_ompC _to_ micF*	-	-	1	-	-	-	-	-	-	-	-	-	-	-	-	-	-	-	-
986977	IS1 (+) +9 bp	*ompF*	-	-	-	-	1	-	-	-	-	-	-	-	-	-	-	-	-	-	-
1979427	IS1 (+) +9 bp	*from_insA5 _to_ uspC*	-	-	-	-	0.84	-	-	-	-	-	-	-	-	-	-	-	-	-	-
3623442	IS1 (+) +9 bp	*yhhH*	-	-	-	-	1	-	-	-	-	-	-	-	-	-	-	-	-	-	-
3803162	IS1 (–) +8 bp	*waaB*	-	-	-	-	0.88	-	-	-	-	-	-	-	-	-	-	-	-	-	-
4531699	IS1 (+) +9 bp	*from_sgcX _to_ yjhP*	-	-	-	-	1	-	-	-	-	-	-	-	-	-	-	-	-	-	-
4360945	IS1 (–) +8 bp	*cadC*	-	-	-	-	-	-	-	-	-	-	0.9	-	1	-	-	-	-	-	-
371758	IS30 (+) +2 bp	*mhpC*	-	-	-	-	-	-	-	-	-	-	-	-	-	-	1	1	1	1	-
1869100	IS1 (+) +8 bp	*yeaH*	-	-	-	-	-	-	-	-	-	-	-	-	-	-	0.3	-	-	-	-
986937	IS5 (–) +4 bp	*ompF*	-	-	-	-	-	-	-	-	-	-	-	-	-	-	-	-	0.17	-	-

1299499 to 1300698	Deletion	*insH21*	1	-	-	1	-	-	-	-	1	-	-	-	-	-	-	-	-	-	-
4092661 to 4104210	Deletion	*[frvA], rhaM, rhaD, rhaA, rhaB, rhaS, rhaR, rhaT, sodA, kdgT, yiiM, [cpxA]*	-	-	1	-	-	-	-	-	-	-	-	-	-	-	-	-	-	-	-
3998113 to 3998206	Deletion	*uvrD*	0.88	-	-	-	-	-	-	-	-	-	-	-	-	-	-	-	-	-	-
4184042 to 4184054	Deletion	*rpoB*	-	-	-	-	1	-	-	-	-	-	-	-	-	-	-	-	-	-	-
4104929 to 4104935	Deletion	*cpxA*	-	-	-	-	-	-	-	-	1	-	-	-	-	-	-	-	-	-	-
2305470 to 2306485	Deletion	*ompC*	-	-	-	-	-	-	-	-	-	-	-	-	-	-	-	-	-	-	1

Table S3 Single-nucleotide polymorphisms in evolved Δ*ampC*

**Position**	**Annotation**	**Referece base**	**Altered base**	**Altered amino acid**	**Frequency**		
					**Δ*ampC*_1_FT**	**Δ*ampC*_2_FT**	**Δ*ampC*_3_FT**
88669	*ftsI*	C	T	Ala257Val	1.00	-	1.00
478037	*acrB*	G	A	Ser608Leu	1.00	-	1.00
4094389	*cpxA*	A	G	Met172Thr	1.00	-	-
3433607	*rpoA*	T	C	Glu261Gly	0.99	-	-
1188957	*mnmA*	T	C	Lys91Lys	0.99	-	-
4387462	*mutL*	T	A	Ser78Arg	0.98	-	-
3528528	*envZ*	G	A	Arg234Cys	0.97	-	1.00
2814783	*alaS*	G	C	Gly196Gly	0.82	-	-
2306322	*from_ompC_to_micF*	CA	C	-	0.82	-	-
4089625	*rhaT*	C	T	Ala277Pro	0.81	-	-
4180206	*yjaZ*	G	A	Ala165Thr	0.81	-	-
1251288	*ycgV*	A	G	Ser41Pro	0.78	-	-
3857063	*from_yidL_to_yidP*	G	A	-	0.76	-	-
833004	*from_ybiC_to_ybiJ*	A	G	-	0.52	-	-
3485056	*from_yhfG_to_ppiA*	C	G	-	0.49	0.35	0.38
1588264	*from_hipB_to_lsrK*	C	T	-	0.47	-	-
89121	*ftsI*	T	C	Ser408Pro	0.43	-	-
579935	*from_appY_to_ompT*	G	GT	-	0.42	-	-
3227077	*from_fadH_to_higA*	G	C	-	0.41	0.44	0.53
3485060	*from_yhfG_to_ppiA*	C	G	-	0.40	-	-

284989	*from_yagI_to_argF*	G	C	-	0.39	0.34	0.30
962370	*msbA*	G	A	Met98Ile	0.34	-	-
1282249	*from_0rI_to_rttR*	A	G	-	0.29	0.25	0.27
1014424	*from_matP_to_ompA*	C	CA	-	0.24	-	-
1481977	*ydcF*	G	A	Thr162Thr	0.23	-	-
93562	*mraY*	G	T	Lys358Asn	0.21	-	-
1304694	*from_kch_to_yciI*	G	A	-	0.20	-	-
3977523	*aslB*	G	A	Met402Ile	0.19	-	-
2512691	*from_yfeD_to_gltX*	A	C	-	0.19	-	-
2811133	*from_yqaB_to_argQ*	A	T	-	0.17	0.19	0.14
353289	*from_codA_to_cynR*	T	C	-	0.17	-	-
198945	*fabZ*	A	C	Thr120Pro	0.16	-	-
776694	*from_lysY_to_lysZ*	T	A	-	0.16	-	-
353286	*from_codA_to_cynR*	T	A	-	0.16	-	-
2514608	*from_valY_to_lysV*	CT	C	-	0.16	-	-
353317	*from_codA_to_cynR*	C	T	-	0.14	-	-
1941441	*ruvC*	C	T	Ala65Thr	0.13	-	-
731810	*from_ybfC_to_ybfD*	A	T	-	0.13	0.12	0.11
1613585	*marR*	C	T	Ala70Val	0.13	-	-
3781445	*envC*	G	A	Trp416*	0.13	-	-
3595746	*ftsE*	G	A	Ala121Val	0.13	-	-
422846	*yajC*	A	G	Tyr35Cys	0.12	-	-
731939	*from_ybfC_to_ybfD*	A	T	-	0.12	0.10	-
353337	*from_codA_to_cynR*	C	T	-	0.12	-	-
2147286	*from_ibsB_to_mdtA*	G	A	-	0.12	0.11	-
3188443	*from_ibsD_to_sibE*	A	G	-	0.12	-	-
2306035	*ompC*	GT	G	Thr65fs	0.11	-	-
353338	*from_codA_to_cynR*	C	A	-	0.11	-	-
477843	*acrB*	C	T	Glu673Lys	0.10	-	-
477976	*acrB*	G	T	Phe628Leu	-	1.00	-
479457	*acrB*	T	G	Ser135Arg	-	1.00	-
2306481	*from_ompC_to_micF*	TTAA	T	-	-	1.00	-
3528023	*envZ*	G	T	Thr402Lys	-	1.00	-
89506	*ftsI*	A	T	Gln536Leu	-	0.99	-
2306477	*from_ompC_to_micF*	A	T	-	-	0.82	-
2222655	*from_yohP_to_dusC*	C	G	-	-	0.60	0.53
2222656	*from_yohP_to_dusC*	A	G	-	-	0.58	0.52
910683	*from_ybjE_to_aqpZ*	G	C	-	-	0.56	0.64
910682	*from_ybjE_to_aqpZ*	T	C	-	-	0.52	-
2222662	*from_yohP_to_dusC*	C	G	-	-	0.51	0.42
910676	*from_ybjE_to_aqpZ*	G	C	-	-	0.42	0.60
2222665	*from_yohP_to_dusC*	C	G	-	-	0.42	0.36
481184	*from_acrA_to_acrR*	AT	A	-	-	0.40	-
481176	*from_acrA_to_acrR*	A	AGGGGG	-	-	0.32	-
1205075	*from_tfaE_to_pinE*	C	G	-	-	0.32	-
4631464	*after_yjtD*	T	TTTTTCAGCTTTTCA	-	-	0.31	-
2730284	*from_bamD_to_raiA*	T	G	-	-	0.22	-
2306477	*from_ompC_to_micF*	A	C	-	-	0.18	-
122568	*aceF*	T	C	Asp129Asp	-	0.16	-
122562	*aceF*	T	G	Val127Val	-	0.15	-

1460401	*from_ynbG_to_insD1*	G	T	-	-	0.15	-
1460395	*from_ynbG_to_insD1*	G	A	-	-	0.14	-
731817	*from_ybfC_to_ybfD*	C	A	-	-	0.14	-
122880	*aceF*	C	A	Ala233Ala	-	0.14	-
3187978	*from_yqiK_to_sibD*	A	G	-	-	0.13	-
3187962	*from_yqiK_to_sibD*	A	C	-	-	0.13	-
1546447	*from_fdnI_to_yddM*	T	C	-	-	0.12	-
2147307	*from_ibsB_to_mdtA*	A	C	-	-	0.12	-
2147312	*from_ibsB_to_mdtA*	A	T	-	-	0.12	-
3187990	*from_yqiK_to_sibD*	G	C	-	-	0.11	-
2147254	*sibB;from_ibsB_to_mdtA*	G	A	-	-	0.11	-
2147247	*sibB;from_ibsB_to_mdtA*	T	C	-	-	0.11	-
3433613	*rpoA*	T	G	Asp259Ala	-	-	0.99
2305858	*ompC*	C	T	Trp124*	-	-	0.96
2711680	*yfiF*	G	C	Arg70Gly	-	-	0.42
1205058	*from_tfaE_to_pinE*	G	GTTTTGCT	-	-	-	0.32
1205055	*from_tfaE_to_pinE*	A	ATT	-	-	-	0.31
1282287	*from_0rI_to_rttR*	T	A	-	-	-	0.31
2170700	*from_gatY_to_fbaB*	T	G	-	-	-	0.22
1892012	*sdaA*	A	C	Asn275Thr	-	-	0.20
1460515	*from_ynbG_to_insD1*	G	C	-	-	-	0.19
3413745	*yhdX*	A	T	Ile85Phe	-	-	0.16
2811104	*from_yqaB_to_argQ*	T	C	-	-	-	0.16
2811097	*from_yqaB_to_argQ*	A	T	-	-	-	0.15
2811130	*from_yqaB_to_argQ*	G	A	-	-	-	0.14
2387247	*nuoL*	G	T	Leu427Ile	-	-	0.11

Table S4 Mutations in *ampC* promoter in evolved WT at different time points during the amoxicillin evolution

Evolved days	R1	R2	R3	R4	R5	R6	R7	R8	R9
1	-	-	-	-	-	-	-	-	-
2	-11 G>A	-11 G>A	-11 G>A	-11 G>A	-11 G>A	-11 G>A	-11 G>A	-	-11 G>A
3	-11 G>A	-11 G>A	-11 G>A	-11 G>A	+54 C>T, +20 G>C, +11 A>C, +7 T>G, +4 C>G, -2 C>G, -11 G>A, -15 C>G, -17A>C	-11 G>A	-11 G>A	-	-11 G>A
6	-11 G>A	-11 G>A	-11 G>A	-11 G>A, +31 del G	-25_-26 ins A, +7 del T	-11 G>A	-11 G>A	-32 A>T	-11 G>A
9	-11 G>A, +35 G>T	-11 G>A, +32 del C	-11 G>A, +37 C>T	-11 G>A, +31 del G	-25_-26 ins A	+32 to +38 del, -32 A>T	-11 G>A, +59 A>G, +56G>T, +54 C>T, +53 T>G	-32 A>T	-32 A>T
12	-11 G>A, +33 C>A +35 G>T	-11 G>A, +32 del C	-11 G>A	-11 G>A, +31 del G	-25_-26 ins A	+32 to +38 del, -32 A>T, -7 G>T, +9A>T, +47_+46 ins TC	-11 G>A	-32 A>T	-32 A>T
16	-11 G>A, +33 C>A	-11 G>A, +32 del C	-11 G>A, +7 del T	-11 G>A, +7 del T, +31 del G	-25_-26 ins A, +7 del T	+32 to +38 del, -32 A>T,	-11 G>A, +23 C>A, +41A>G, +55T>A	-32 A>T, +29 to +32 del	-32 A>T

						+47_+46 ins TC			
22	-11 G>A, +33 C>A	-11 G>A, +32 del C	-11 G>A	-11 G>A, +31 del G	-25_-26 ins A	+32 to +38 del,<br>-32 A>T,<br>+47_+46 ins TC	-11 G>A,<br>+23 C>A	-32 A>T	-32 A>T

Table S5 Primers and probes used in this study

Primers/Probes	Sequence
ampC_F	ACTGGGGTTGTTTCCGGGTG
ampC_R	TGGTTGAGTTTGAGTGGCTG
gadph_F	GCTGAAGGCGAAATGAAAGG
gadph_R	GTACCAGGATACCAGTTTCACG
F-AmpC Prom	CGCCGGTGATGATCCTGCTGGT
R-AmpC prom	ATGTTTTGCTGACCGAACCTAA
ampC_probe	/56-FAM/ATTAACTGCCTGCAACACCACGC/36-TAMSp/
gadph_probe	/56-FAM/CTGGACGTGGTTGAAGGTATGCAGT/36-TAMSp/
